# Exogenous norepinephrine attenuates the efficacy of sunitinib in a mouse cancer model

**DOI:** 10.1186/1756-9966-33-21

**Published:** 2014-02-20

**Authors:** Guo-Hua Deng, Jie Liu, Jie Zhang, Ying Wang, Xing-Chen Peng, Yu-Quan Wei, Yu Jiang

**Affiliations:** 1 Cancer Center, State Key Laboratory of Biotherapy, West China Hospital of Sichuan University, Chengdu, Sichuan Province 610041, China

**Keywords:** Tumor, Chronic stress, Norepinephrine, Sunitinib, Propranolol

## Abstract

**Background:**

Sunitinib alone exhibits satisfactory efficacy in several mouse homografts and xenografts but unsatisfactory efficacy in many kinds of solid tumors in clinic. Different from animals, receiving a diagnosis of cancer impacts chronic stress on patients. Here, we examine whether norepinephrine (**NE**), one of the most potent stress related hormones, leads to the difference in the efficacy of sunitinib between clinical and preclinical trials.

**Methods:**

The influence of NE on mouse melanoma B16F1 cells under sunitinib was evaluated in vitro and in vivo. The β-AR/cAMP/PKA (β-adrenoceptor/cyclic adenosine monophosphate/protein kinase A) signaling pathway was also evaluated in human lung adenocarcinoma cells.

**Results:**

We found that NE upregulated the expression of VEGF, IL-8 and IL-6 in vitro and stimulated tumor growth in vivo, which was mediated by β-AR/cAMP/PKA signaling pathway and could be inhibited by propranolol, a β-blocker for hypertension for decades.

**Conclusions:**

This research indicates exogenous norepinephrine attenuates the efficacy of sunitinib, and a combination of sunitinib and propranolol might be suggested as a new strategy in solid tumor in clinic.

## Background

Psychosocial factors including chronic stress, depression, dejection, and lack of social support have been proved risk factors for cancer occurrence and progression by psychological and epidemiological studies [1-4]. It is well known that chronic stress impacts on immune system, neuroendocrine system, lymphatic and hematopoietic system. Stress inhibits the immune response ability in antigen-specific T-cells and natural killer cells while stimulates the secretion of proinflammatory cytokines, such as IL-1, IL-2, IL-6, IL-8, IL-11 and TNF-α, which were regarded as co-factors for modulating the growth and progression of tumor [5,6]. Recent studies reported that chronic stress can also immediately affect the growth, development and metastasis of malignant tumors via hormone receptors on tumor cells [7-10].

In mammals under stress, an increased level of stress-related hormone (**SRH**) can be induced by the activation of the hypothalamic-pituitary-adrenal and the sympathetic-adrenal medullary axes. Activation by stress on sympathetic nervous system results in the release of catecholamines from the adrenal medulla and sympathetic nerve terminals [6,10]. Catecholamines consist of several kinds of substances such as dopamine, histamine, serotonin, epinephrine and norepinephrine (**NE**). The last one is regarded as the most potential SRH related to tumors in mammals [10,11]. As ligands, catecholamines can bind adrenergic receptors (**ARs**) coupled with G-protein which can be classified as several subtypes such as α1, α2, β1, β2 and β3 ARs. Many types of ARs locate on tumor cells, providing the theory that chronic stress impacts on the progression of cancer. Furthermore, the effect of stress could be mimicked with NE or β2-AR agonists, and abolished with surgical sympathetic denervation, β-AR antagonists or knocking down β2-AR gene by small interfering RNA [6,10,12].

It is accepted that a solid tumor can not progress without angiogenesis. VEGF, one of the most important angiogenic factors, can recruit and induce endothelial cells to proliferate and migrate, thereby starting the critical step of tumor expansion. Previous studies have demonstrated that NE upregulates VEGF, IL-8, IL-6 and MMP expression levels in some kinds of tumor cells *in vitro* such as melanoma, breast cancer, colon cancer, prostate cancer, ovary cancer, pancreatic cancer and nasopharynx cancer. Besides, migration of cancer cells can be stimulated by NE, which can be blocked by nonselective β-AR antagonist, propranolol [7-9,13-18]. In mouse models *in vivo*, chronic stress stimulates the growth, progression and metastasis of tumors, which can also be inhibited by propranolol [13-15,19]. The clinical research reported that propranolol lowered the rate of breast cancer-specific mortality, cancer recurrence and distant metastasis, thus improved relapse-free survival and cancer specific survival [20-22].

Tumor angiogenesis plays a key role in development of solid tumors. Sunitinib, one kind of anti-angiogenic drugs, is a tyrosine kinase inhibitor with the ability of blocking VEGFR1, VEGFR2, VEGFR3, PDGFRα, PDGFRβ, c-Kit and RET. It can induce tumor cell death and inhibit tumor proliferation and vascularization [23-25]. However, in clinic, treatment with sunitinib alone is of poor curative effect or even inefficacious for many types of solid tumors. On the contrary, sunitinib exhibits satisfactory efficacy in mouse homografts of melanoma, Lewis lung cancer, renal cancer and colon cancer, and xenografts of human colorectal cancer *in vivo*[24,26-28]. Additionally, monotherapy with anti-angiogenic drugs including endostatin and bevacizumab also shows the discrepancy between clinical and preclinical results [29,30]. Thus the question should be presented: Why does the difference of the curative response between the human and animal occur?

Different from tumor-bearing mice, receiving a diagnosis of malignancy and battling with chronic uncertainties as regards treatment, progression, recurrence, and mortality is a major chronic stressor imaginable for patients with cancer. Given the impact of chronic stress on a cancer patient, the confluence of the psychological and physical discomfort places the patient at high risk for the occurrence of stress-induced behavioral alterations which usually presents depression, anxiety, sadness, fear and hopelessness [4,11,31,32]. We reported previously that 39.5% of cancer patients were unwilling to realize the diagnosis of cancer, 63.0% were burdened with mental stress and 33.0% considered the impact of mental stress above that of somatic symptoms [33].

We hypothesize that the discrepancy of the efficacy of anti-angiogenic drugs between clinical and preclinical results is caused by chronic stress, which has not been yet identified. So in this research, the goal is to investigate whether NE, one of the most potent stress related hormones, can attenuate the efficacy of sunitinib in a mouse model and whether this effect can be blocked by propranolol.

## Materials and methods

### Cell culture

The murine melanoma B16F1 cells and human lung adenocarcinoma A549 cells, kind gifts from State Key Laboratory of Biotherapy (Sichuan University, Chengdu), were authenticated by the supplier [29] and cultured in RPMI 1640 complete medium containing 10% fetal bovine serum (FBS), 100 U/mL penicillin, and 100 μg/mL streptomycin at 37°C with 5% CO2 in humidified atmosphere.

### Reagents

NE, 3-(4,5-dimethylthiazol-2-yl)-2,5 diphenyltetrazolium bromide (**MTT**), dimethylsulfoxide (**DMSO**), isoproterenol, dobutamine and terbutaline were purchased from Sigma (St. Louis, MO, USA); propranolol and 8-CPT from Enzo (Germany); forskolin from Biovision (USA); H-89 and myristoylated PKI from Calbiochem (USA); sunitinib from Pfizer (USA); RNAiso plus and One Step SYBR® PrimeScript™ RT-PCR Kit from TaKaRa (Japan).

### *In vitro* cell proliferation assays for measuring the IC_50_ (half maximal inhibitory concentration) of sunitinib in B16F1 cells

B16F1 cells were harvested and seeded in 96-well plates (5,000 cells/200 μL complete medium/ well). After 24 hours incubation, the cells were exposed to various concentrations (0–100 μM, each concentration had six replicate wells) of sunitinib for 48 h. Following sunitinib treatment, 20 μL of 5 mg/mL MTT was added to each well and incubated at 37°C for 4 hours. The plates were centrifuged, the supernatants were carefully discarded and formazan crystals were dissolved in 150 μL DMSO. At last, the light absorbance at 490 nm was determined in a luminescence plate reader (PerkinElmer, USA) according to the manufacturer’s instructions.

### Evaluation of the influence of NE on mRNA and protein expression *in vitro*

B16F1 and A549 cells were dispensed in six-well culture plates (2 × 10^5^/well). After incubation overnight, 2 mL complete RPMI 1640 medium was replaced by serum-free medium for 24 hours to make the cells adapt serum-starvation. Then cells were incubated in 2 mL renewed serum-free medium containing 0, 0.1, 1, 10 μM NE or 10 μM NE +10 μM propranolol (propranolol was added 30 minutes prior to NE). Culture supernatants were gathered and cells were homogenized in RNAiso plus at different time points designed for detection by ELISA (3, 6, 12 and 24 hours) and real-time PCR (1, 2, 3 and 4 hours), respectively. In addition, we evaluated the influence of 10 μM NE in B16F1 cells treated with sunitinib at the concentration equal to **IC**_**50**_ (sunitinib was added 30 minutes following NE)_**.**_

### Evaluation of β-AR (β-adrenoceptor)/cAMP/PKA signaling pathway

A recent study identified that the β2-AR/cAMP/PKA signaling pathway mediated the up-regulation of VEGF by NE on human ovarian cancer cells [9]. Here we tested the role of this pathway on A549 cells. First, 10 μM α-AR antagonist phentolamine and 10 μM β-AR antagonist propranolol were added into the cell cultures 30 minutes before adding 10 μM NE in order to assess the role of AR subtypes (α-AR VS β-AR). Second, A549 cells were incubated in serum-free medium containing 10 μM β-AR agonist isoproterenol, 10 μM β1-AR agonist dobutamine, 10 μM β2-AR agonist terbutaline, 100 μM selective activator of the cAMP receptor 8-CPT, 10 μM adenylate cyclase agonist forskolin, 100 μM cAMP-dependent protein kinase inhibitor H-89 or 10 μM myristoylated protein kinase inhibitor PKI. Similar to propranolol, H-89 or PKI was added 30 minutes before the addition of 10 μM NE [17]. Culture supernatants were harvested 6 hours after treatment for ELISA and cells were homogenized in RNAiso plus 2 hours after treatment for RT-PCR. In order to evaluate the proliferation and migration of A549 cells under the inhibitors PKI and H-89, MTT assay and scratch wound healing assay were performed as previously described [34-36].

### *In vivo* tumor model

C57BL6 female mice (4–6 weeks old) were purchased from the Laboratory Animal Center of Sichuan University. Male mice should be excluded for possible stress from mates in the cage. The animal experiments with the C57BL6 mice were consistent with protocols approved by the Institutional Animal Care and Treatment Committee of Sichuan University. The mice were maintained under pathogen-free conditions with food and water *ad libitum*, on 12 h/12 h day/night cycle, a temperature of 21–25°C, three mice per cage.

B16F1 cells were trypsinized, centrifuged and then resuspended in serum-free medium. For implantation, tumors cells were subcutaneously inoculated in the right flanks of mice (5 × 10^5^ cells per mouse). Tumor measurements were made periodically with manual calipers every three days, and tumor volume was calculated applying the formula: π/6 × length × width^2^. At the end of the test, mice were sacrificed and tumors were excised, weighed and photographed. The serum from mice was harvested.

### Establishment of chronic stress *in vivo* and treatment with sunitinib

Eight days after inoculation when the tumors reached an average diameter of 5 mm, mice were randomly assigned to four groups each consisting of six mice. The mice were narcotized by chloral hydrate i.p. (4%, 10 mL/kg) and then microosmotic pumps (Alzet model 1004, Durect, Cupertino, CA) were implanted subcutaneously on the left back of the mice for the establishment of chronic stress. The microosmotic pumps implanted in the body could keep functional and pump drugs contained continuously for up to 4 weeks. The pumps were filled with 100 μL normal saline containing 56 mM NE, 56 mM propranolol or both of them at a dose of 1 μmol/100 g/day [14]. Ascorbic acid (0.2%) was added as a preservative into every pump. The pumps full of just normal saline and ascorbic acid were used in the control group. The initiation of treatment with sunitinib by oral gavage (80 mg/kg/day [27]) was on the next day. The animals were sacrificed after 14 days of treatment.

### ELISA

The concentrations of VEGF, IL-8 and IL-6 proteins in culture supernatants or serum were detected using mouse or human ELISA Kits (Neobioscience, Beijing) following the manufacturer’s protocol. The light absorbance at 450 nm was read in a luminescence plate reader (PerkinElmer, USA)**.** The values of concentrations were calculated by interpolation from a standard curve. Each experiment was repeated at least three times in duplicate.

### Immunohistochemistry for CD31, VEGF, β1-AR and β2-AR

Immunohistochemical studies were performed as previously described [26] using antibodies against CD31 (rat antimouse monoclonal antibody, diluted 1:300; BD Pharmingen, San Diego, CA, USA), VEGF (rabbit antimouse polyclonal antibody, diluted 1:200; Bioss Biotechology, Beijng), β1-AR & β2-AR (rabbit antimouse polyclonal antibody, diluted 1:300; Bioss Biotechology, Beijng). CD31 was stained on the frozen sections from B16F1 tumors for measuring microvessel density (**MVD**), VEGF on the formalin-fixed and paraffin-embedded sections from B16F1 tumors for comparing the expression levels among four groups and β1-AR and β2-AR on the slides of B16F1 cells for detecting the status of β-ARs in cells. Phosphate buffered saline was used instead of the primary antibody for negative controls.

### Assessment of microvessel density

MVD was assessed by choosing three areas with thickest microvessel distribution (hot spot) according to immunoreactivity for CD31 at low microscopic magnification (40 ×) and then counting the number of immunoreactive endothelial cells and microvessels from three 200 × high power fields in hot pot areas [37,38].

### RT-PCR analysis

The assessment of VEGF, IL-8 and IL-6 gene expression was conducted using semiquantitative real-time reverse transcription-PCR (**RT-PCR**). Total RNA from A549 cells was isolated with RNAiso plus according to the RNA extraction protocols. Then the RNA was separated by 1% agarose gel electrophoresis and visualized by golden view to test the quality and integrity of RNA samples using the Gel Doc image system (Bio-Rad, Hercules, CA, USA). RT-PCR was conducted using One Step SYBR® PrimeScript™ RT-PCR Kit (Perfect Real Time) and amplified with CFX 96™ Real-Time System in C1000™ Thermal Cycler (Bio-Rad, USA). Glyceraldehyde-3-phosphate dehydrogenase (**GAPDH**) was applied as an internal positive control. The primers in this study were as follows: GAPDH: sense 5′- ACCACAGTCCATGCCATCAC -3′, antisense 5′- TCCACCACCCTGTTGCTGTA -3′; VEGF: sense 5′- TGGATCCATGAACTTTCTGCTGTC -3′, antisense 5′- TCACCGCCTTGGCTTGTCACAT -3′; IL-8: sense 5′-CTTTGTCCATTCCCACTTCTGA-3′, antisense 5′-TCCCTAACGGTTGCCTTTGTA T-3′; IL-6: sense 5′- ATGAACTCCTTCTCCACAAGCGC -3′, antisense 5′- GAAGAGCCCTCAGGCTGGACTG -3′ [12,39-41]. The PCR cycler condition was according to the recommendations in the manufacturer’s instructions. Reactions were performed in a 25-μL volume and each sample was run at least in duplicate. The levels of expression of VEGF, IL-8, and IL-6 mRNA in each sample were normalized to the GAPDH mRNA level. The relative expression of VEGF, IL-8, and IL-6 mRNA was calculated applying the comparative C_T_ method [18,39].

### Statistical analysis

The data are expressed as the mean ± SD. Changes in protein and mRNA levels of VEGF, IL-8 and IL-6, the averaged tumor volume and weight were calculated by one way analysis of variance (ANOVA) with an LSD post-hoc test and an unpaired student’ t test using SPSS, version 15.0 (SPSS, Chicago, IL). A p value less than 0.05 was considered as statistically significant.

## Results

### NE upregulates VEGF, IL-8, and IL-6 protein levels in culture supernatants of B16F1 (with or without sunitinib) and A549 cells, which can be blocked by propranolol

**Figure 1:**
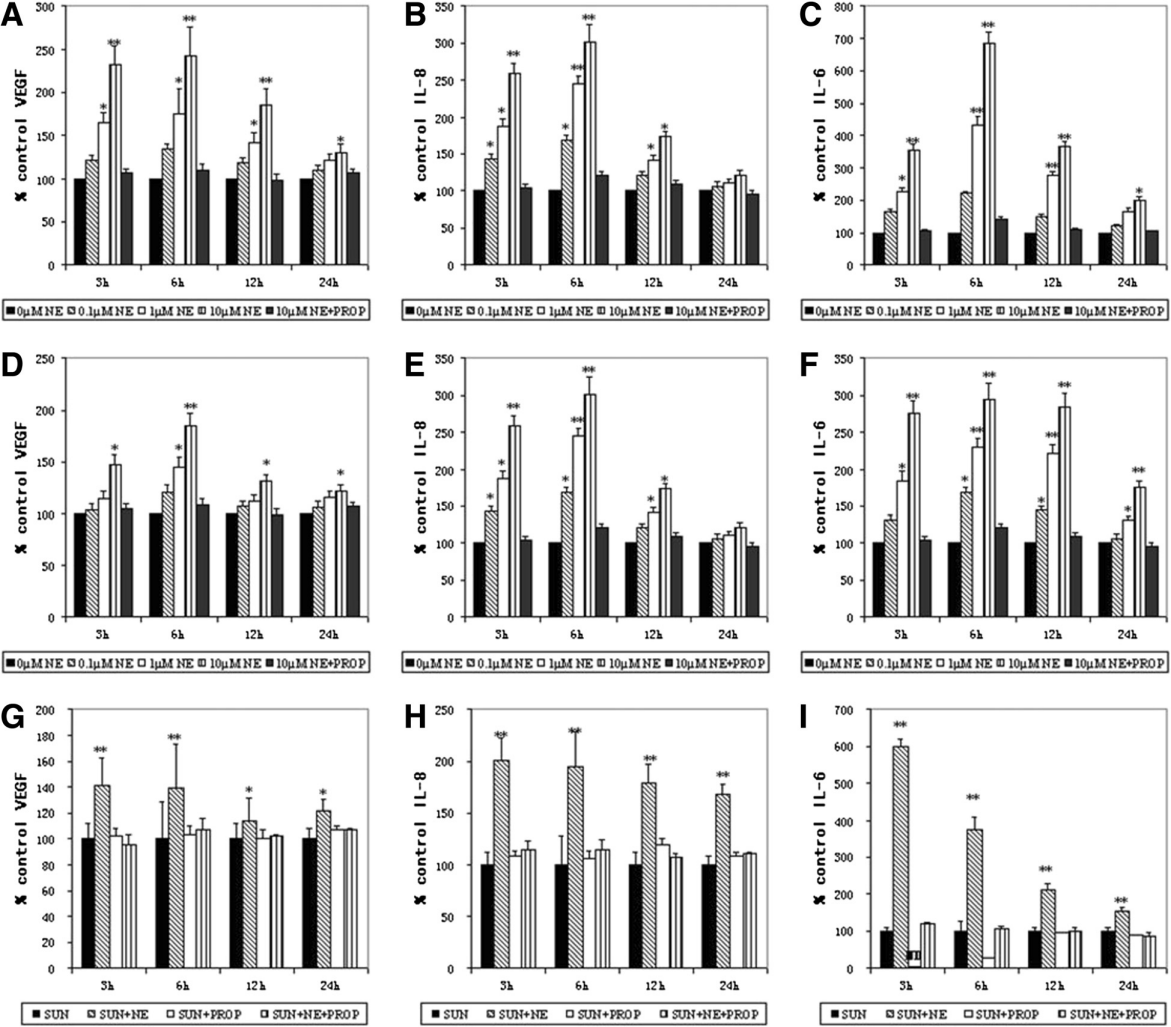
**Effect of NE in vitro (with or without sunitinib).** VEGF, IL-8 and IL-6 protein levels in culture supernatants by A549 **(A, B, and C)** and B16F1 **(D, E and F)** cells were measured after incubation with 0 (CON), 0.1, 1, 10 μM NE and 10 μM NE + 10 μM PROP for 3, 6, 12 and 24 hours. The levels of VEGF, IL-8, and IL-6 protein in B16F1 **(G, H and I)** cells incubated with 3.35 μM SUN alone (CON), 3.35 μM SUN + 10 μM NE, 3.35 μM SUN + 10 μM PROP and 3.35 μM SUN + 10 μM NE + 10 μM PROP for 6 hours were also detected. Data are represented as percentage of the control well, which was set as 100% in each experimental series. All bars represent the mean ± SD of at least three experiments performed in duplicate. CON, control. SUN, sunitinib. ND, not detectable. *, P ≤ 0.05; **, P ≤ 0.001.

A NE dose-dependent and time-dependent increase in VEGF, IL-8 and IL-6 protein levels in culture supernatants of both B16F1 and A549 cells with a peak increase at the 6 hours time point and 10 μM concentration, which could be blocked by 10 μM propranolol (Figure 1A-F). In A549 cells, treatment with 10 μM NE for 6 h caused a remarkable increase to 242.79 ± 19.86%, 331.56 ± 24.41% and 685.85 ± 34.72% (P < 0.001) of control levels for VEGF, IL-8 and IL-6 protein levels, respectively (Figure 1A-C). Likewise, in B16F1 cells, VEGF, IL-8 and IL-6 protein levels arrived at 185.15 ± 12.13%, 301.35 ± 24.98% and 294.40 ± 23.17% (P < 0.001) of control levels in response to exposure to 10 μM NE for 6 hours (Figure 1D-F). Overall, the increase could be most seen in both two cells at the NE concentration ranging from 0.1 to 10 μM since 3 hours after treatment. However, as time went on, the extent of the increase reduced 6 hours later.

In addition, the IC_50_ of sunitinib in B16F1 cells measured by cell proliferation assays was 3.35 μM. The results about B16F1 cells treated with sunitinib at the concentration equal to IC_50_ indicated that NE could also upregulate VEGF, IL-8, and IL-6 proteins with a peak increase at the 6 hours time, which could also be blocked by 10 μM propranolol (Figure 1G-I).

### NE promotes tumor growth in the murine B16F1 model under the treatment of sunitinib and can be blocked by propranolol

**Figure 2:**
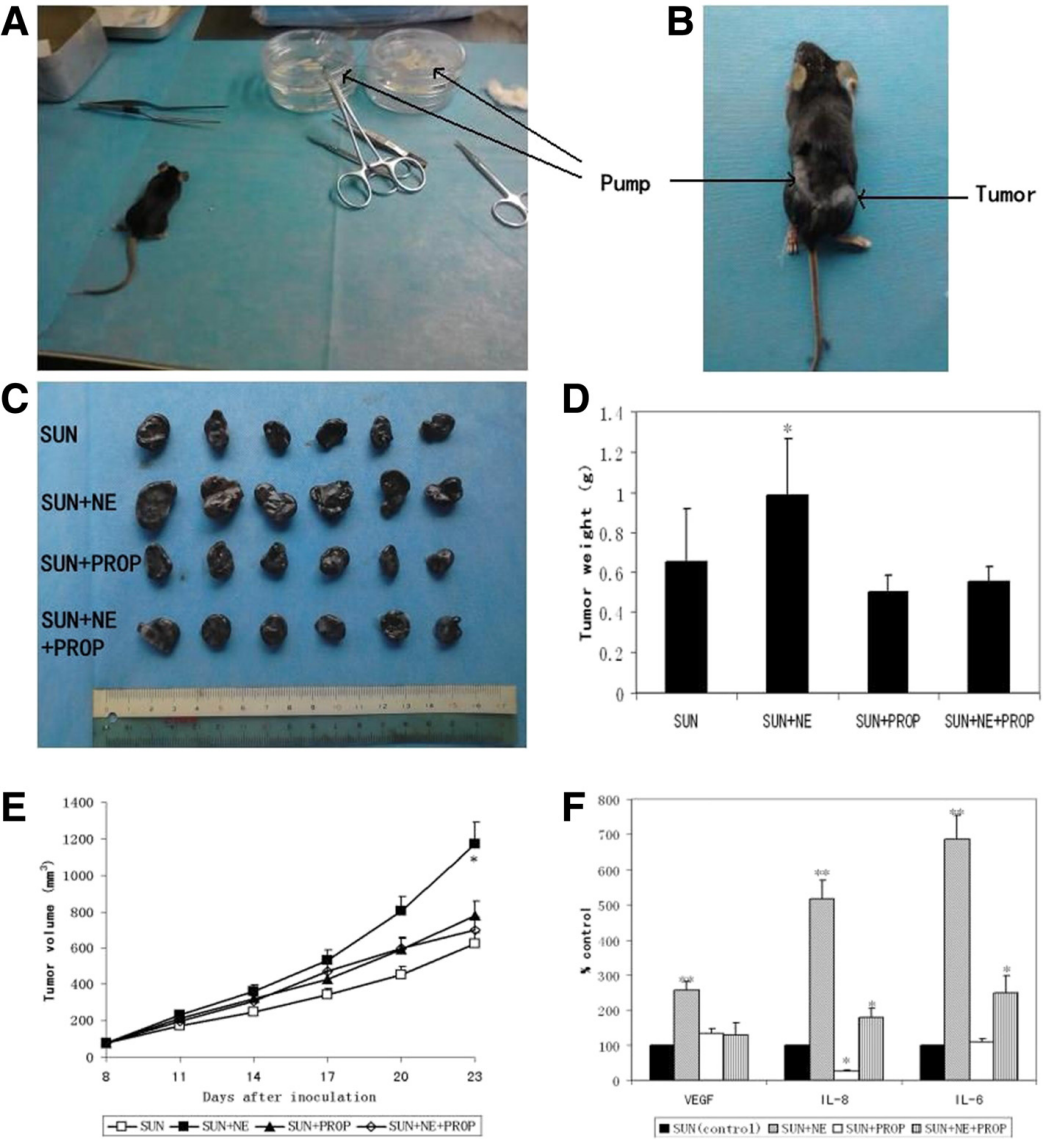
**NE attenuates the efficacy of sunitinib *in vivo*. A)** Preoperative preparation for implanting micro-osmotic pumps which should soaked in normal saline for at least 48 hours at 37°C. **B)** The pumps were implanted subcutaneously on the left back of the mice. **C)** The photograph of the tumors excised from all mice in 4 groups in B16F1 models. **D)** The bar chart showing the weight of the tumors. **E)** The line chart showing tumor growth curves. **F)** VEGF, IL-8 and IL-6 protein levels measured by ELISA in the serum from the mice in B16F1 models. Data are represented as percentage of the control (SUN without NE or PROP). All bars represent the mean ± SD. SUN, sunitinib. PROP, propranolol. *, P ≤ 0.05; **, P ≤ 0.001.

Our results showed that NE speeded up the tumor growth rate in the B16F1 model treated with sunitinib. Similar with the results *in vitro* as above, the effect of NE could be blocked by propranolol (P < 0.05) (Figure 2A-E). NE increased the tumor weight by 51.65% compared with normal saline (0.99 ± 0.28 g VS 0.65 ± 0.27 g, P = 0.014) and 79.22% compared with the combination of NE and propranolol (0.99 ± 0.28 g VS 0.55 ± 0.08 g, P = 0.002) (Figure 2D).

As shown in Figure 2F, VEGF, IL-8 and IL-6 protein levels tested by the ELISA assay were upregulated by NE in the serum from the B16F1 model, which could be blocked by propranolol. NE increased VEGF, IL-8 and IL-6 protein levels by 155.77%, 417.77% and 586.21% compared with normal saline, respectively (P < 0.001).

### NE stimulates tumor angiogenesis in the B16F1 model treated with sunitinib

**Figure 3:**
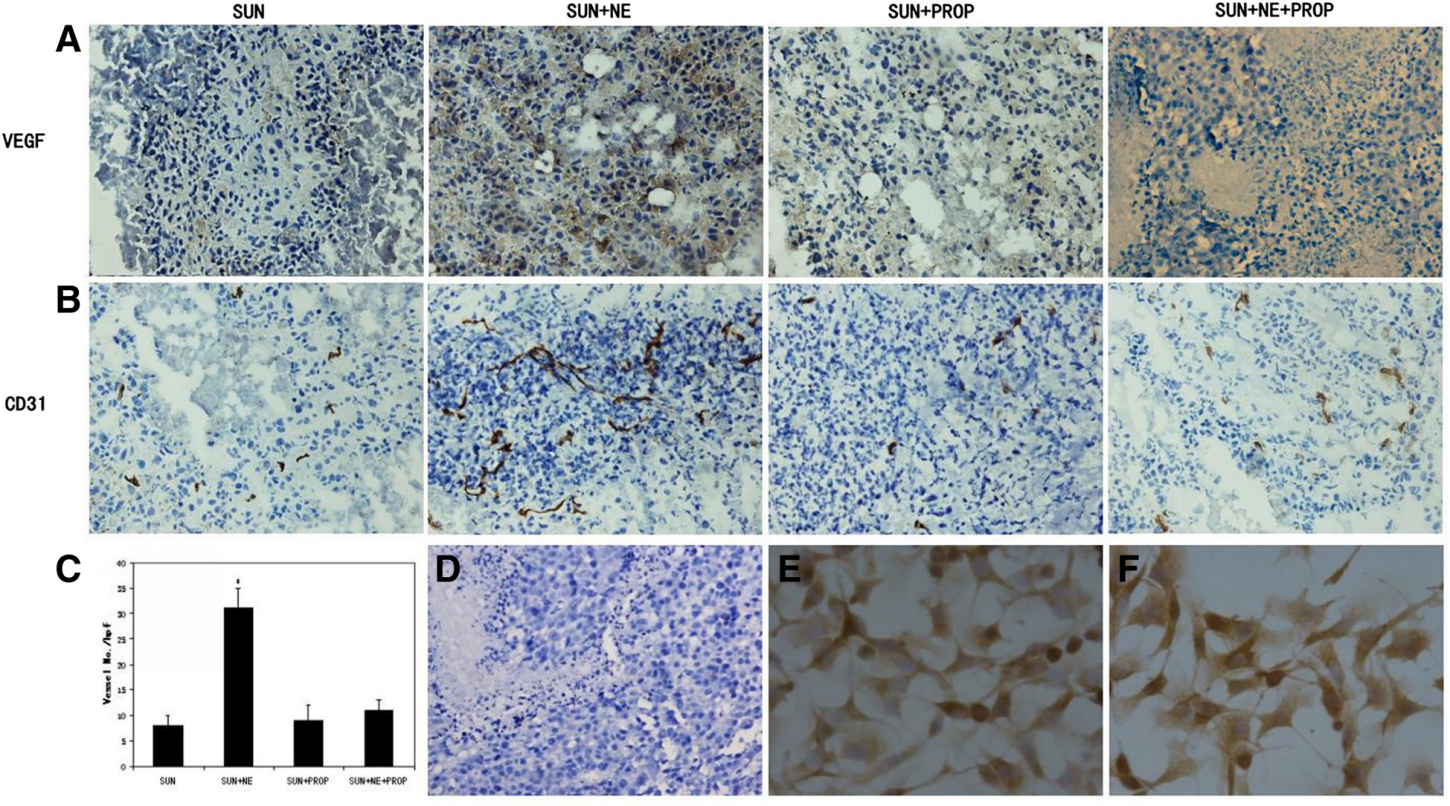
**NE promotes angiogenesis *in vivo*. A)** Representative photographs of the B16F1 tumor sections examined by immunohistochemical staining for VEGF (× 200 magnification). **B)** Immunohistochemical staining for CD31 (× 200 magnification). **C)** The bar chart showing MVD calculated by CD31 immunoreactivity. Each bar represents the average vessel number of each group, expressed as the mean ± SD. *, P ≤ 0.05. **D)** The photograph of immunohistochemical staining in negative control slides for VEGF. **E)** Immunohistochemical staining for β1-AR on the slides of B16F1 cells (× 200 magnification). **F)** Immunohistochemical staining for β2-AR (× 200 magnification).

Immunohistochemical staining for VEGF on the formalin-fixed and paraffin-embedded sections showed a much stronger staining in the tumors of the group stimulated by NE than the other three groups (normal saline, propranolol and NE + propranolol) (Figure 3A). There is no brown or yellow staining in negative control slides for VEGF wherein no primary antibodies were used (Figure 3D).

Similar to VEGF, the significant increase in MVD, detected by immunohistochemical staining for CD31 on frozen sections, occurred in the tumors of the mice treated with sunitinib and stimulated by NE (P < 0.05) (Figure 3B-C).

### Beta1-AR and β2-AR are expressed in B16F1 cells

Immunohistochemical staining for β1-AR and β2-AR on the slides of B16F1 cells was utilized to evaluate the status of β-AR via which NE affected cells. The results showed strong β1 and β2-AR immunoreactivivty located in the cytoplasma (Figure 3E, F, respectively). The staining was invisible in negative control slides (not shown).

### NE upregulates VEGF, IL-8, and IL-6 gene expression in A549 cells

**Figure 4:**
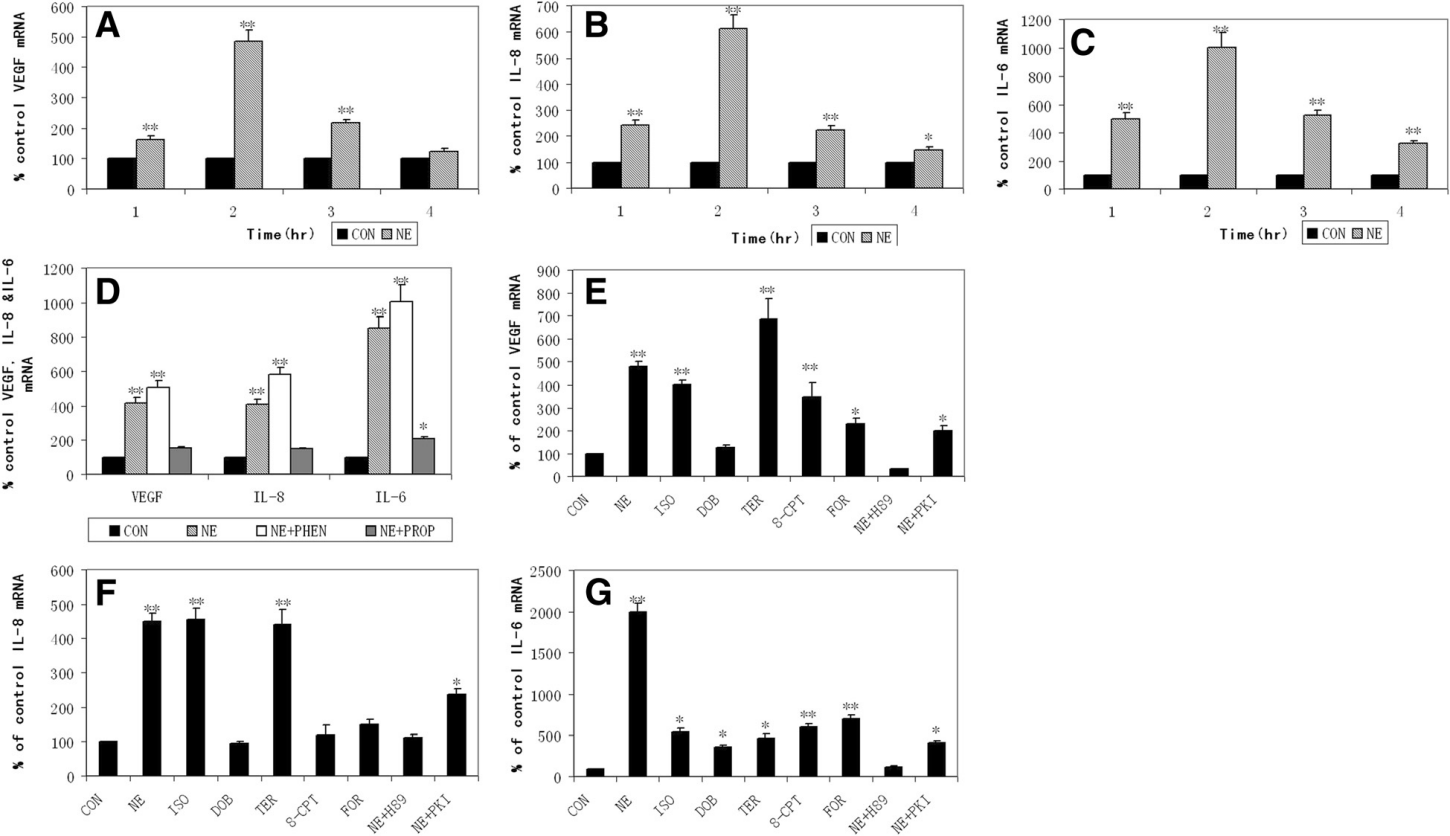
**Evaluation of β-AR/cAMP/PKA signaling pathway by RT-PCR.** The NE-dependent stimulation of VEGF **(A)**, IL-8 **(B)**, and IL-6 **(C)** mRNA levels with a peak at 2 hours was observed in treatment of A549 cells with 10 μM NE **(A, B, and C)**. This effect could not be blocked by phentolamine (PHEN) **(D)**. Representative results of VEGF **(E)**, IL-8 **(F)**, and IL-6 **(G)** mRNA levels treated with NE, isoproterenol (ISO), dobutamine (DOB), terbutaline (TER), 8-CPT, forskolin (FOR), NE + H89 or NE + PKI for 2 hours. Values are presented as percent of untreated control levels. Each bar represents the mean ± SD. ND, not detectable. *, P ≤ 0.05; **, P ≤ 0.001.

Although the up-regulation of VEGF, IL-8, and IL-6 protein levels by NE was described as above, we assessed the effect of NE on the expression of these three genes to further clarify the mechanism concerning the modulation of these three proteins in A549 cells. The results indicated that the levels of VEGF, IL-8, and IL-6 mRNA increased rapidly with a peak after 2 hours of treatment and decreased gradually thereafter in A549 cells exposed to 10 μM NE (Figure 4A-C).

### Beta-AR/cAMP/PKA signaling pathway contributes to the NE effect in A549 cells

For determining whether β-AR mediated the NE effect, phentolamine (α-AR antagonist) was used here to contrast with propranolol. We observed that, opposite to propranolol, phentolamine could not abrogate the NE-induced increase of VEGF, IL-8, and IL-6 mRNA levels in A549 cells (Figure 4D).

Isoproterenol (nonselective β-AR agonist), dobutamine (selective β1-AR agonist) and terbutaline (selective β2-AR agonist) upregulated VEGF, IL-8, and IL-6 mRNA levels, which indicated that both β1-AR and β2-AR mediated the NE-dependent effect (Figure 4E-G). Moreover, comparing with β1-AR, β2-AR played a key role as a mediator special for the NE-induced stimulation of VEGF and IL-8 gene expression in A549 cells because terbutaline had a higher degree of up-regulation than dobutamine. Additionally, 8-CPT and forskolin (cAMP analogs) both raised VEGF, IL-8, and IL-6 mRNA levels implicating cAMP as a mediator. Lastly, H-89 (PKA inhibitor) nearly checked the effect of NE which could be just partially inhibited by PKI.

**Figure 5:**
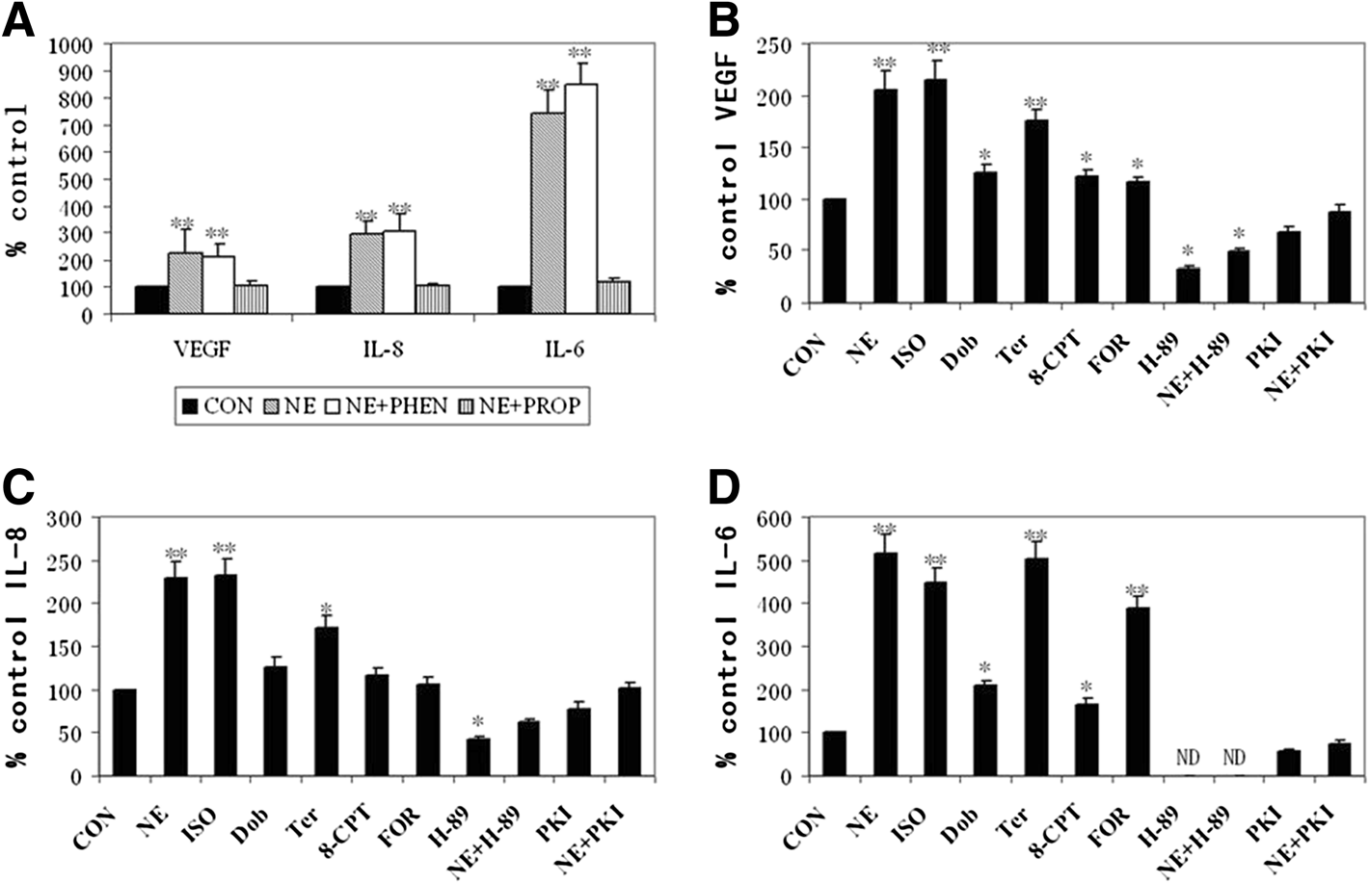
**Evaluation of β-AR/cAMP/PKA signaling pathway by ELISA.** The NE-dependent stimulation of VEGF, IL-8, and IL-6 protein levels could not be blocked by phentolamine (PHEN) **(A)**. Representative results of VEGF **(B)**, IL-8 **(C)**, and IL-6 **(D)** protein levels treated with NE, isoproterenol (ISO), dobutamine (DOB), terbutaline (TER), 8-CPT, forskolin (FOR), NE + H89 or NE + PKI for 6 hours. Values are presented as percent of untreated control levels. Each bar represents the mean ± SD. *, P ≤ 0.05; **, P ≤ 0.001.

To further identify the role of β-AR/cAMP/PKA signaling pathway in NE-treated A549 cells, the changes in VEGF, IL-8, and IL-6 protein levels tested by the ELISA assay related to mRNA levels as above were also analyzed. We observed similar changes in VEGF, IL-8, and IL-6 protein levels with their mRNA levels (Figure 5A-D).

**Figure 6:**
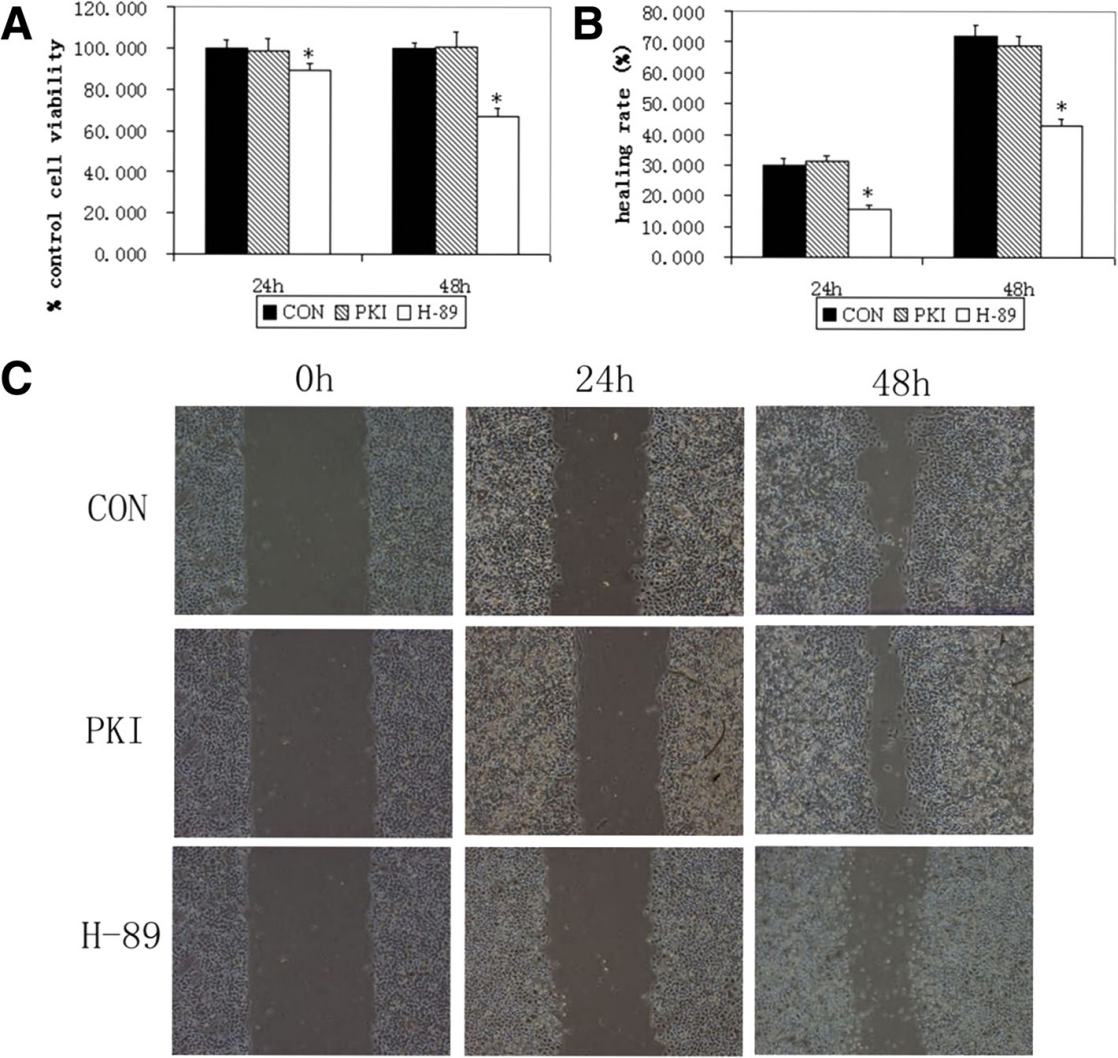
**The proliferation and migration of A549 cells under PKI and H-89.** MTT assay showed that H-89 inhibited the proliferation of A549 cells **(A)** and wound healing assay showed H-89 lowered the migration of A549 cells **(B and C)**. CON, control. *, P ≤ 0.05.

We also evaluated the proliferation and migration of A549 cells under the inhibitors PKI and H-89. The results showed that, different from PKI, H-89 inhibited the proliferation (Figure 6A) and migration (Figure 6B-C) of A549 cells. These results were consistent with the protein and gene levels of VEGF, IL-8 and IL-6 of A549 cells under PKI and H-89.

## Discussion

In this study we showed that NE spurred tumor growth in the murine melanoma model treated with sunitinib by gavage *in vivo* and could be inhibited by propranolol. We also identified that NE upregulated VEGF, IL-8, and IL-6 protein levels in B16F1 cells in the presence or absence of the treatment with sunitinib at the concentration equal to IC_50,_ which was blocked by propranolol. In addition, NE-dependent up-regulation in both protein and gene levels of VEGF, IL-8, and IL-6 was observed in human lung adenocarcinoma cells in which β-AR/cAMP/PKA signaling pathway was proved as the important mechanism. Chronic stress has been acknowledged as an important factor affecting patients with cancer and the effect of chronic stress may be persistent during the process from diagnosis for cancer to death of cancer. The activation on sympathetic nervous system by stress gives rise to the increased level of catecholamines resulting in several biological effects via ARs such as VEGF-caused stimulation in angiogenesis, raised levels of cytokines including IL-8 and IL-6 [42]. These effects were also proved in our study and found as at least a part of factors attenuating the efficacy of sunitinib in preclinical models.

In order to mimic chronic stress in patients, a wide variety of stress models in animals were established, e.g. addition of corticosterone to drinking water, transfer to a cold room at 4°C, subcutaneously administration with NE or β2-AR agonists, restraint procedure using open-ended Plexiglas cylindrical restrainers, social defeat, social isolation, unpredictable chronic mild stress, repeated social defeat, subcutaneous microosmotic pumps containing NE [12,43-49]. However, some of stress models aforementioned have limitations more or less and thus induce unpredictable impacts on tests *in vivo*. For addition of corticosterone to drinking water, this test might not control the volume of water drunk by animals and thus the reliable uptake of corticosterone can not be evaluated especially when uptake of water was interrupted by the disorders in animals such as a heavy tumor burden [49]. For the restraint test, it was found in our laboratory that mice would adapt the open-ended Plexiglas cylindrical restrainers in the later stage. So the restraint test might not sustain enough stress if the observation in a test *in vivo* should be kept for a long time [45]. Seeing that microosmotic pumps (1004 type) are of the ability of pumping drugs contained incessantly for up to 4 weeks and exhibit reliable effects in mouse models, the pumps were taken into account in our research to deal with the short half life period of NE. It is well known that in clinic patients are under chronic stress after diagnosed with cancer prior to treatment. Thereby, in order to mimic patients in clinic as possible, sunitinib was administrated 30 minutes following NE in tests *in vitro*, and treatment with sunitinib was started 1 day after the implantation of pumps containing NE in tests *in vivo*.

Tumor neovascularization or angiogenesis is closely related with proangiogenic factors such as VEGF, IL-8, IL-6, TGF and TNF released by tumor cells and immune cells. In analogy to tumors cells, lymphocytes and macrophages in the tumor microenviroment also express β-ARs triggered by NE with the following increased levels of VEGF, IL-8, and IL-6 [50-53]. The NE-induced up-regulation of VEGF, IL-8, and IL-6 protein levels was found in a number of human cancer cell lines such as colon cancer, nasopharyngeal cancer, ovarian cancer, prostate cancer and melanoma [7,8,13,17,18]. This effect of NE was identified in murine melanoma B16F1 cells and human lung adenocarcinoma A549 cells in our study. In addition, this phenomenon was also observed in murine colon cancer CT26 cells and some human cancer cells (e.g., nasopharyngeal cancer HNE1 & CNE2 cells, breast cancer MDA-MB-231 & MDA-MB-468 cells and colon cancer HT-29 & SW480 cells) in other studies in our laboratory (unpublished date not shown). However, to our knowledge, nothing is known of the influence of NE in cancer cells treated with sunitinib *in vitro*. Our date indicated that, in B16F1 cells treated with sunitinib at IC_50_ concentration, NE also increased VEGF, IL-8, and IL-6 protein expression in culture supernatants, which could be inhibited by propranolol. This result offered at least a mechanism for the difference in the efficacy of sunitinib between clinical and preclinical trials. It should be considered if sunitinib acts via some of its targets on B16 cells. Previous studies reported that B16 cells did not express VEGFR1, VEGFR2, VEGFR3 [54,55], PDGFRα and PDGFRβ [56] but no more than 10% of B16 cells expressed c-Kit [57]. Whether sunitinib acts on B16 cells through the c-Kit target remains to be investigated in the further study. Chronic stress has been demonstrated to promote development and progression of tumors in several human cancer cells in xenografts including prostate cancer, ovarian cancer and breast cancer [9,13,15,46,58], whereas no date regarding the influence of chronic stress in cancer models under sunitinib *in vivo* has been reported so far. This study showed that consecutive NE pumped stimulated the growth of primary tumor in a mouse melanoma model and could be blocked by propranolol. This result provided a piece of evidence for the discrepancy in the efficacy of sunitinib between clinical and preclinical trials and was consistent with the results in the other studies in our laboratory (mouse colon cancer CT26 homograft and human colon cancer SW480 and HT-29 xenografts, unpublished date not shown).

To further investigate stress-induced angiogenesis *in vivo*, we analysed the immunoreactivity for VEGF and CD31, counted the MVD and measured the protein levels of VEGF, IL-8 and IL-6 in mouse serums. As expected, in accordance with the results *in vivo* as mentioned in the previous paragraph, chronic stress promoted angiogenesis and neovascularization in B16F1 tumors, thus withstood the anti-angiogenic treatment of sunitinib. Interestingly, relatively low VEGF expression was found in tumor and endothelial cells while stronger VEGF expression usually found in peri-necrotic tumors cells mainly by reason of hypoxia as reported in the other study [59]. In clinic, the serum levels of VEGF, IL-8 and IL-6 have been suggested as potentially predictive markers for survival in cancer patients under sunitinib. Bauerschlag et al. [60] found that 18 cases with a decrease in VEGF serum concentration out of 29 ovarian cancer patients with sunitinib therapy had a longer progression-free survival (**PFS**) compared to 11 cases with an increase in VEGF serum concentration (10.5 VS 2.9 months). Likewise, the lower serum VEGF level was reported to be associated with longer PFS and objective response rate in patients under sunitinib with bevacizumab-refractory metastatic renal cancer [61]. Bellmunt et al. [62] announced that the low serum IL-8 level was related to long median time to progression in urothelial cancer patients receiving sunitinib as first-line treatment. Comparing with healthy donors, an increased level of IL-8 was detected in serums from medullary thyroid carcinoma patients with distant metastases [63]. Zhu et al. [64] reported that advanced hepatocellular carcinoma patients with high serum levels of IL-8 and IL-6 were of high mortality and rapid tumor progression after sunitinib. On the other hand, patients with a decrease level of IL-6 had better PFS and overall survival. Additionally, during sunitinib treatment, a more elevated IL-6 level was in correspondence with higher hazard of mortality or immediate progression.

ARs are a family of G protein-coupled receptors, also called serpentine receptors whose ligands mainly include chemokines and neurotransmitters [31]. Since the expression of β-ARs was observed in human lung adenocarcinoma A549 cells [65,66], only an immunohistochemical analysis for β-ARs in B16F1 cells was carried out. Hegener et al. [65] also found that the internalization and endocytosis of β2-AR in A549 cells were stimulated by terbutaline (selective β2-AR agonist) and forskolin (cAMP analogs), whereas blocked by propranolol. In our study, the strong expression of β-ARs located in the cytoplasma and there was no difference of staining intensity between β1-AR and β2-AR discerned with naked eyes. This finding in our study provided the basis for following research on the β-AR/cAMP/PKA pathway in B16F1 cells. Considering ARs play a key role mediating the effect on tumors induced by chronic stress and endow tumor cells the potential to respond to neurotransmitters, few scholars suggest the receptor-based interference of intracellular ARs signaling pathway as a new approach to resist this effect [9,42,67,68]. Powe et al. [69] found, in breast cancer, β2-AR strongly immunoreactive in cases with a luminal phenotype and good clinic outcome while α1b-AR and α2c-AR over-expressed in basal-like phenotypes of poor prognosis. So ARs might be supposed to be potential predictors for survival and probable indicators for targeted therapy with AR blockers.

In the present research, it was approved in A549 cells that the NE-induced up-regulation in both protein and gene levels of VEGF, IL-8 and IL-6 was chiefly mediated by β-AR/cAMP/PKA signaling pathway which had been found to play a key role in mouse xenografts of melanoma and ovarian cancer [9,17]. The stimulation of β-ARs by neurotransmitters induces multiple signaling pathways of which the most important one approved is cAMP/PKA/**CREB** (cAMP response element binding protein). Then the activation of CREB, a transcription factor, initiates the arachidonic acid cascade, the Src/STAT and the EGFR pathways followed by a wide variety of biological effects [9,70].

## Conclusions

Taken together, our data support the hypothesis that exogenous norepinephrine gives rise to the attenuation in the efficacy of sunitinib in a mouse melanoma model and provide a reason for the discrepancy of the efficacy of anti-angiogenic drugs between clinical and preclinical results. The combination of anti-angiogenic agents with β-AR antagonists such as propranolol, a drug for cardio-vascular disease for decades, might eliminate the ineffectiveness of anti-angiogenic agents alone and enhance their efficacy in some types of tumors, which has yet to be approved in prospective randomized controlled trials in clinic.

## Competing interests

The authors declare no conflict of interests.

## Authors’ contributions

YJ and YQW designed the procedure of the study. GHD carried out the plan and drafted the manuscript. JL, JZ and YW participated in cell culture, animal experiments and immunohistological analysis. XCP assisted in RT-PCR and statistical analysis. YJ and YQW supervised the whole experimental work and revised the manuscript. All authors read and approved the manuscript.
